# Dioxidobis{2-[(*E*)-*p*-tolyl­imino­meth­yl]phenolato}molybdenum(VI)

**DOI:** 10.1107/S1600536810032836

**Published:** 2010-08-21

**Authors:** Mehdi Hatefi, Iran Sheikhshoaie, Valiollah Mirkhani, Majid Moghadam, Reza Kia

**Affiliations:** a Chemistry Department, Shahid Bahonar University, Kerman, Iran; b Department of Chemistry, University of Isfahan, Isfahan 81746-73441, Iran; c Department of Chemistry, Science and Research Branch, Islamic Azad University, Tehran, Iran; d X-ray Crystallography Laboratory, Plasma Physics Research Center, Science and Research Branch, Islamic Azad University, Tehran, Iran

## Abstract

The asymmetric unit of the title compound, [Mo(C_14_H_12_NO)_2_O_2_], comprises half of the complex with the full mol­ecule generated by the application of twofold symmetry. The Mo^VI^ atom is surrounded by two oxide O atoms and the two sets of *N*,*O*-donor atoms of the bidentate Schiff base ligands. The resulting N_2_O_4_ donor set defines a distorted octa­hedral coordination geometry. Inter­molecular C—H⋯O contacts link mol­ecules into chains along the *b* axis. The crystal structure is further stabilized by inter­molecular π–π inter­actions [ring centroid–centroid distance = 3.724 (6) Å].

## Related literature

For related structures with MoO_2_ units and for the synthesis, see: Arnaiz *et al.* (2000 ▶); Holm *et al.* (1996 ▶); Syamal & Maurya (1989 ▶).
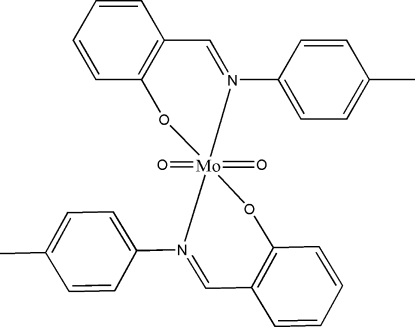


## Experimental

### 

#### Crystal data

[Mo(C_14_H_12_NO)_2_O_2_]
*M* *_r_* = 548.43
Monoclinic, 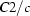

*a* = 26.375 (8) Å
*b* = 6.8095 (8) Å
*c* = 15.648 (10) Å
β = 116.94 (2)°
*V* = 2505.4 (18) Å^3^
*Z* = 4
Mo *K*α radiation
μ = 0.56 mm^−1^
*T* = 296 K
0.21 × 0.11 × 0.08 mm

#### Data collection

Stoe IPDS II diffractometer
Absorption correction: multi-scan [*MULABS* in *PLATON*  (Spek, 2009 ▶; Blessing, 1995 ▶)] *T*_min_ = 0.892, *T*_max_ = 0.957
4305 measured reflections
2006 independent reflections
761 reflections with *I* > 2σ(*I*)
*R*_int_ = 0.144

#### Refinement

*R*[*F* ^2^ > 2σ(*F* ^2^)] = 0.079
*wR*(*F* ^2^) = 0.168
*S* = 0.83
2006 reflections
124 parameters
H-atom parameters constrained
Δρ_max_ = 0.55 e Å^−3^
Δρ_min_ = −0.81 e Å^−3^

### 

Data collection: *X-AREA* (Stoe & Cie, 2005 ▶); cell refinement: *X-AREA*; data reduction: *X-AREA*; program(s) used to solve structure: *SHELXTL* (Sheldrick, 2008 ▶); program(s) used to refine structure: *SHELXTL*; molecular graphics: *SHELXTL*; software used to prepare material for publication: *SHELXTL* and *PLATON* (Spek, 2009 ▶).

## Supplementary Material

Crystal structure: contains datablocks global, I. DOI: 10.1107/S1600536810032836/tk2696sup1.cif

Structure factors: contains datablocks I. DOI: 10.1107/S1600536810032836/tk2696Isup2.hkl

Additional supplementary materials:  crystallographic information; 3D view; checkCIF report

## Figures and Tables

**Table 1:** Hydrogen-bond geometry (Å, °)

*D*—H⋯*A*	*D*—H	H⋯*A*	*D*⋯*A*	*D*—H⋯*A*
C9—H9*A*⋯O2^i^	0.93	2.34	3.237 (18)	163
C13—H13*A*⋯O2^ii^	0.93	2.42	3.164 (17)	136

Symmetry codes: (i) 
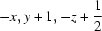
; (ii) 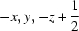.

**Fig. 1.**
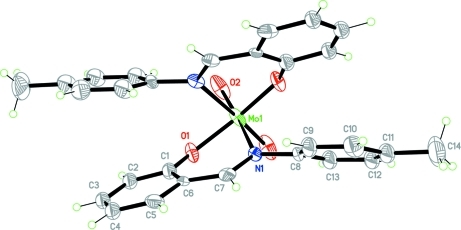
The molecular structure of the title complex showing the atom-labelling scheme and displacement ellipsoids at the 35% probability level. Symmetry code for the unlabeled atoms: -x, y, 1/2 - z.

**Fig. 2.**
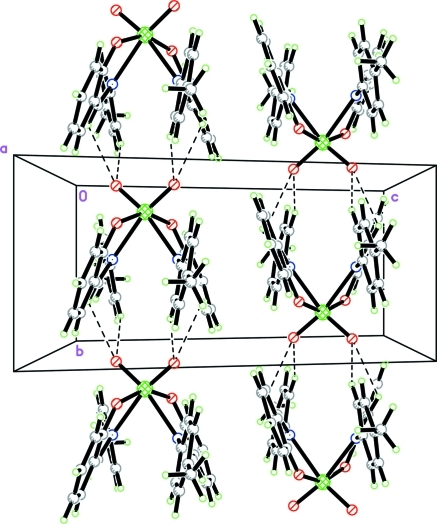
A view of the crystal packing of the title compound down the a axis showing connections of molecules through C—H···O interactions (shown as dashed lines) along the b axis.

## supplementary crystallographic information

### Comment

Numerous chemical reactions are catalyzed by complexes containing the dioxomolybdenum(VI) unit, MoO_2_ (Arnaiz *et al.* 2000). Moreover, Schiff base compounds containing molybdenum play a significant role in the chemistry of molybdoenzymes (Holm *et al.* 1996; Syamal & Maurya, 1989).

The asymmetric unit of the title compound, Fig. 1, comprises half of the complex. The Mo atom is located on a crystallographic 2-fold axis. The Mo^VI^ atom is surrounded by two oxo-O atoms and the N_2_O_2_ donor atoms of two bidentate Schiff base ligands to define a distorted octahedral coordination geometry. Intermolecular C—H···O contacts link molecules into chains along the *b* axis, Table 1 and Fig. 2. The crystal structure is further stabilized by intermolecular π–π interactions with the ring centroid(C1– C6) to centroid(C8–C13)^i^ distance being 3.724 (6) Å for *i*: -*x*, *y*, 1/2-*z*.

### Experimental

The title complex was prepared by adding MoO_2_(acac)_2_ and the ligand (molar ratio 1:1) to dry methanol (30 ml), followed by refluxing for 1 h. Small, light-yellow crystals were filtered off and recrystallized from acetonitrile. The quality of the crystal was not optimal and it was weakly diffracting. Although recrystallization was attempted repeatedly, better crystals were not obtained.

### Refinement

All H atoms were positioned geometrically (C–H = 0.93–0.96 Å) and constrained to refine with the parent atoms with *U*_iso_ (H) = 1.2–1.5 *U*_eq_ (C).

### Crystal data

**Table:**

[Mo(C_14_H_12_NO)_2_O_2_]	*F*(000) = 1120
*M**_r_* = 548.43	*D*_x_ = 1.454 Mg m^−^^3^
Monoclinic, *C*2/*c*	Mo *K*α radiation, λ = 0.71073 Å
Hall symbol: -C 2yc	Cell parameters from 2560 reflections
*a* = 26.375 (8) Å	θ = 1.7–29.6°
*b* = 6.8095 (8) Å	µ = 0.56 mm^−^^1^
*c* = 15.648 (10) Å	*T* = 296 K
β = 116.94 (2)°	Block, colourless
*V* = 2505.4 (18) Å^3^	0.21 × 0.11 × 0.08 mm
*Z* = 4	

### Data collection

**Table:**

Stoe IPDS II diffractometer	2006 independent reflections
Radiation source: fine-focus sealed tube	761 reflections with *I* > 2σ(*I*)
graphite	*R*_int_ = 0.144
Detector resolution: 0.15 mm pixels mm^-1^	θ_max_ = 25.5°, θ_min_ = 2.6°
φ and ω scans	*h* = −31→22
Absorption correction: multi-scan (*MULABS* in *PLATON*; Blessing, 1995)	*k* = −7→8
*T*_min_ = 0.892, *T*_max_ = 0.957	*l* = −18→18
4305 measured reflections	

### Refinement

**Table:**

Refinement on *F*^2^	Primary atom site location: structure-invariant direct methods
Least-squares matrix: full	Secondary atom site location: difference Fourier map
*R*[*F*^2^ > 2σ(*F*^2^)] = 0.079	Hydrogen site location: inferred from neighbouring sites
*wR*(*F*^2^) = 0.168	H-atom parameters constrained
*S* = 0.83	*w* = 1/[σ^2^(*F*_o_^2^) + (0.0476*P*)^2^] where *P* = (*F*_o_^2^ + 2*F*_c_^2^)/3
2006 reflections	(Δ/σ)_max_ < 0.001
124 parameters	Δρ_max_ = 0.55 e Å^−^^3^
0 restraints	Δρ_min_ = −0.81 e Å^−^^3^

### Special details

**Table:**

Geometry. All e.s.d.'s (except the e.s.d. in the dihedral angle between two l.s. planes) are estimated using the full covariance matrix. The cell e.s.d.'s are taken into account individually in the estimation of e.s.d.'s in distances, angles and torsion angles; correlations between e.s.d.'s in cell parameters are only used when they are defined by crystal symmetry. An approximate (isotropic) treatment of cell e.s.d.'s is used for estimating e.s.d.'s involving l.s. planes.
Refinement. Refinement of *F*^2^ against ALL reflections. The weighted *R*-factor *wR* and goodness of fit *S* are based on *F*^2^, conventional *R*-factors *R* are based on *F*, with *F* set to zero for negative *F*^2^. The threshold expression of *F*^2^ > σ(*F*^2^) is used only for calculating *R*-factors(gt) *etc*. and is not relevant to the choice of reflections for refinement. *R*-factors based on *F*^2^ are statistically about twice as large as those based on *F*, and *R*- factors based on ALL data will be even larger.

### Fractional atomic coordinates and isotropic or equivalent isotropic displacement parameters (Å^2^)

**Table:**

	*x*	*y*	*z*	*U*_iso_*/*U*_eq_	
Mo1	0.0000	−0.2892 (2)	0.2500	0.0502 (7)	
O1	−0.0423 (3)	−0.2223 (11)	0.3209 (4)	0.053 (2)	
O2	−0.0486 (4)	−0.4409 (11)	0.1680 (6)	0.079 (3)	
N1	0.0559 (5)	−0.0212 (13)	0.3428 (5)	0.037 (3)	
C1	−0.0533 (6)	−0.061 (2)	0.3573 (8)	0.0479 (18)	
C2	−0.1029 (5)	−0.0625 (17)	0.3693 (7)	0.0479 (18)	
H2A	−0.1260	−0.1733	0.3541	0.057*	
C3	−0.1165 (5)	0.1058 (16)	0.4047 (7)	0.0479 (18)	
H3A	−0.1493	0.1052	0.4127	0.057*	
C4	−0.0846 (5)	0.2697 (19)	0.4278 (6)	0.0479 (18)	
H4A	−0.0957	0.3798	0.4503	0.057*	
C5	−0.0345 (5)	0.2726 (19)	0.4176 (6)	0.054 (4)	
H5A	−0.0118	0.3845	0.4333	0.065*	
C6	−0.0187 (5)	0.1007 (18)	0.3827 (6)	0.037 (3)	
C7	0.0360 (5)	0.1081 (16)	0.3804 (6)	0.041 (3)	
H7A	0.0589	0.2167	0.4087	0.049*	
C8	0.1129 (6)	0.013 (2)	0.3551 (7)	0.044 (3)	
C9	0.1308 (6)	0.198 (2)	0.3415 (7)	0.063 (2)	
H9A	0.1063	0.3049	0.3253	0.075*	
C10	0.1856 (6)	0.219 (2)	0.3524 (7)	0.067 (4)	
H10A	0.1972	0.3429	0.3429	0.081*	
C11	0.2243 (6)	0.063 (2)	0.3771 (8)	0.063 (2)	
C12	0.2048 (6)	−0.115 (2)	0.3891 (7)	0.063 (2)	
H12A	0.2294	−0.2217	0.4054	0.075*	
C13	0.1500 (5)	−0.144 (2)	0.3781 (7)	0.063 (2)	
H13A	0.1384	−0.2690	0.3861	0.075*	
C14	0.2838 (6)	0.095 (3)	0.3862 (11)	0.120 (6)	
H14A	0.3085	−0.0083	0.4239	0.181*	
H14B	0.2984	0.2193	0.4169	0.181*	
H14C	0.2821	0.0955	0.3236	0.181*	

### Atomic displacement parameters (Å^2^)

**Table:**

	*U*^11^	*U*^22^	*U*^33^	*U*^12^	*U*^13^	*U*^23^
Mo1	0.0830 (15)	0.0203 (8)	0.0809 (12)	0.000	0.0666 (11)	0.000
O1	0.056 (6)	0.046 (5)	0.080 (5)	0.015 (5)	0.050 (5)	0.018 (5)
O2	0.110 (10)	0.039 (5)	0.138 (8)	−0.036 (6)	0.099 (7)	−0.043 (5)
N1	0.045 (9)	0.033 (6)	0.037 (6)	0.027 (6)	0.022 (6)	0.014 (4)
C1	0.043 (5)	0.055 (4)	0.049 (4)	0.005 (4)	0.024 (4)	0.007 (3)
C2	0.043 (5)	0.055 (4)	0.049 (4)	0.005 (4)	0.024 (4)	0.007 (3)
C3	0.043 (5)	0.055 (4)	0.049 (4)	0.005 (4)	0.024 (4)	0.007 (3)
C4	0.043 (5)	0.055 (4)	0.049 (4)	0.005 (4)	0.024 (4)	0.007 (3)
C5	0.064 (10)	0.060 (9)	0.034 (6)	0.006 (9)	0.018 (6)	−0.006 (6)
C6	0.031 (9)	0.064 (9)	0.017 (6)	0.010 (7)	0.012 (6)	0.002 (6)
C7	0.053 (11)	0.037 (7)	0.029 (6)	0.010 (7)	0.015 (7)	0.018 (5)
C8	0.029 (9)	0.075 (10)	0.034 (7)	0.010 (8)	0.020 (6)	0.004 (6)
C9	0.053 (6)	0.079 (5)	0.062 (4)	0.031 (6)	0.031 (4)	0.015 (4)
C10	0.072 (11)	0.063 (9)	0.073 (8)	−0.010 (10)	0.038 (8)	−0.020 (8)
C11	0.053 (6)	0.079 (5)	0.062 (4)	0.031 (6)	0.031 (4)	0.015 (4)
C12	0.053 (6)	0.079 (5)	0.062 (4)	0.031 (6)	0.031 (4)	0.015 (4)
C13	0.053 (6)	0.079 (5)	0.062 (4)	0.031 (6)	0.031 (4)	0.015 (4)
C14	0.070 (14)	0.165 (18)	0.150 (14)	−0.014 (12)	0.071 (11)	−0.031 (12)

### Geometric parameters (Å, °)

**Table:**

Mo1—O2	1.694 (9)	C5—H5A	0.9300
Mo1—O2^i^	1.694 (9)	C6—C7	1.459 (14)
Mo1—O1^i^	1.950 (6)	C7—H7A	0.9300
Mo1—O1	1.950 (6)	C8—C13	1.385 (15)
Mo1—N1	2.382 (9)	C8—C9	1.394 (17)
Mo1—N1^i^	2.382 (9)	C9—C10	1.384 (15)
O1—C1	1.330 (12)	C9—H9A	0.9300
N1—C7	1.293 (12)	C10—C11	1.400 (16)
N1—C8	1.446 (14)	C10—H10A	0.9300
C1—C6	1.367 (15)	C11—C12	1.363 (17)
C1—C2	1.405 (16)	C11—C14	1.528 (18)
C2—C3	1.388 (14)	C12—C13	1.391 (16)
C2—H2A	0.9300	C12—H12A	0.9300
C3—C4	1.344 (14)	C13—H13A	0.9300
C3—H3A	0.9300	C14—H14A	0.9600
C4—C5	1.402 (14)	C14—H14B	0.9600
C4—H4A	0.9300	C14—H14C	0.9600
C5—C6	1.432 (14)		
			
O2—Mo1—O2^i^	104.8 (6)	C4—C5—H5A	120.5
O2—Mo1—O1^i^	98.2 (3)	C6—C5—H5A	120.5
O2^i^—Mo1—O1^i^	98.2 (4)	C1—C6—C5	119.7 (12)
O2—Mo1—O1	98.2 (4)	C1—C6—C7	123.9 (11)
O2^i^—Mo1—O1	98.2 (3)	C5—C6—C7	116.3 (12)
O1^i^—Mo1—O1	153.0 (4)	N1—C7—C6	126.5 (11)
O2—Mo1—N1	167.6 (4)	N1—C7—H7A	116.7
O2^i^—Mo1—N1	87.6 (3)	C6—C7—H7A	116.7
O1^i^—Mo1—N1	79.2 (3)	C13—C8—C9	119.4 (13)
O1—Mo1—N1	80.2 (3)	C13—C8—N1	118.7 (12)
O2—Mo1—N1^i^	87.6 (3)	C9—C8—N1	121.9 (12)
O2^i^—Mo1—N1^i^	167.6 (4)	C10—C9—C8	118.9 (14)
O1^i^—Mo1—N1^i^	80.2 (3)	C10—C9—H9A	120.5
O1—Mo1—N1^i^	79.2 (3)	C8—C9—H9A	120.5
N1—Mo1—N1^i^	80.0 (4)	C9—C10—C11	122.9 (14)
C1—O1—Mo1	136.7 (7)	C9—C10—H10A	118.6
C7—N1—C8	116.2 (10)	C11—C10—H10A	118.6
C7—N1—Mo1	122.2 (9)	C12—C11—C10	116.2 (14)
C8—N1—Mo1	121.5 (7)	C12—C11—C14	123.3 (15)
O1—C1—C6	122.9 (12)	C10—C11—C14	120.5 (14)
O1—C1—C2	116.5 (12)	C11—C12—C13	123.1 (14)
C6—C1—C2	120.6 (12)	C11—C12—H12A	118.4
C3—C2—C1	118.0 (12)	C13—C12—H12A	118.4
C3—C2—H2A	121.0	C8—C13—C12	119.5 (13)
C1—C2—H2A	121.0	C8—C13—H13A	120.2
C4—C3—C2	123.4 (12)	C12—C13—H13A	120.2
C4—C3—H3A	118.3	C11—C14—H14A	109.5
C2—C3—H3A	118.3	C11—C14—H14B	109.5
C3—C4—C5	119.2 (12)	H14A—C14—H14B	109.5
C3—C4—H4A	120.4	C11—C14—H14C	109.5
C5—C4—H4A	120.4	H14A—C14—H14C	109.5
C4—C5—C6	119.0 (12)	H14B—C14—H14C	109.5
			
O2—Mo1—O1—C1	−136.1 (11)	C2—C1—C6—C5	−3.0 (15)
O2^i^—Mo1—O1—C1	117.6 (11)	O1—C1—C6—C7	−5.2 (16)
O1^i^—Mo1—O1—C1	−9.2 (10)	C2—C1—C6—C7	174.4 (9)
N1—Mo1—O1—C1	31.5 (11)	C4—C5—C6—C1	1.9 (14)
N1^i^—Mo1—O1—C1	−50.1 (11)	C4—C5—C6—C7	−175.7 (8)
O2—Mo1—N1—C7	62 (2)	C8—N1—C7—C6	−176.2 (9)
O2^i^—Mo1—N1—C7	−119.5 (8)	Mo1—N1—C7—C6	7.8 (13)
O1^i^—Mo1—N1—C7	141.7 (8)	C1—C6—C7—N1	10.1 (16)
O1—Mo1—N1—C7	−20.8 (7)	C5—C6—C7—N1	−172.5 (9)
N1^i^—Mo1—N1—C7	59.9 (7)	C7—N1—C8—C13	137.6 (10)
O2—Mo1—N1—C8	−113.4 (19)	Mo1—N1—C8—C13	−46.3 (11)
O2^i^—Mo1—N1—C8	64.6 (8)	C7—N1—C8—C9	−44.5 (13)
O1^i^—Mo1—N1—C8	−34.2 (7)	Mo1—N1—C8—C9	131.6 (9)
O1—Mo1—N1—C8	163.4 (8)	C13—C8—C9—C10	−0.7 (15)
N1^i^—Mo1—N1—C8	−116.0 (8)	N1—C8—C9—C10	−178.7 (8)
Mo1—O1—C1—C6	−25.2 (17)	C8—C9—C10—C11	−0.3 (16)
Mo1—O1—C1—C2	155.2 (7)	C9—C10—C11—C12	0.8 (16)
O1—C1—C2—C3	−178.3 (9)	C9—C10—C11—C14	178.8 (10)
C6—C1—C2—C3	2.1 (16)	C10—C11—C12—C13	−0.2 (17)
C1—C2—C3—C4	0.0 (16)	C14—C11—C12—C13	−178.1 (11)
C2—C3—C4—C5	−1.0 (15)	C9—C8—C13—C12	1.3 (15)
C3—C4—C5—C6	0.1 (14)	N1—C8—C13—C12	179.3 (9)
O1—C1—C6—C5	177.4 (9)	C11—C12—C13—C8	−0.9 (16)

Symmetry codes: (i) −*x*, *y*, −*z*+1/2.

### Hydrogen-bond geometry (Å, °)

**Table:**

*D*—H···*A*	*D*—H	H···*A*	*D*···*A*	*D*—H···*A*
C9—H9A···O2^ii^	0.93	2.34	3.237 (18)	163
C13—H13A···O2^iii^	0.93	2.42	3.164 (17)	136

Symmetry codes: (ii) −*x*, *y*+1, −*z*+1/2; (iii) −*x*, *y*, −*z*+1/2.
